# Recent advances in understanding the phenotypes of osteoarthritis

**DOI:** 10.12688/f1000research.20575.1

**Published:** 2019-12-12

**Authors:** Ali Mobasheri, Simo Saarakkala, Mikko Finnilä, Morten A. Karsdal, Anne-Christine Bay-Jensen, Willem Evert van Spil

**Affiliations:** 1 Department of Regenerative Medicine, State Research Institute Centre for Innovative Medicine, Vilnius, 08661, Lithuania; 2 Research Unit of Medical Imaging, Physics and Technology, University of Oulu, Oulu, FI-90014, Finland; 3 Centre for Sport, Exercise and Osteoarthritis Research Versus Arthritis, Queen’s Medical Centre, Nottingham, UK; 4 ImmunoScience, Nordic Bioscience Biomarkers and Research, Herlev, DK-2730, Denmark; 5 Division of Internal Medicine & Dermatology, Department of Rheumatology & Clinical Immunology, University Medical Center Utrecht, Utrecht, The Netherlands; 6 Rheumatology, Dijklander Hospital, 1620 AR Hoorn, The Netherlands

**Keywords:** osteoarthritis, molecular endotype, clinical phenotype, imaging, biomarker, patient stratification, clinical trials, drug development

## Abstract

Recent research in the field of osteoarthritis (OA) has focused on understanding the underlying molecular and clinical phenotypes of the disease. This narrative review article focuses on recent advances in our understanding of the phenotypes of OA and proposes that the disease represents a diversity of clinical phenotypes that are underpinned by a number of molecular mechanisms, which may be shared by several phenotypes and targeted more specifically for therapeutic purposes. The clinical phenotypes of OA supposedly have different underlying etiologies and pathogenic pathways and they progress at different rates. Large OA population cohorts consist of a majority of patients whose disease progresses slowly and a minority of individuals whose disease may progress faster. The ability to identify the people with relatively rapidly progressing OA can transform clinical trials and enhance their efficiency. The identification, characterization, and classification of molecular phenotypes of rapidly progressing OA, which represent patients who may benefit most from intervention, could potentially serve as the basis for precision medicine for this disabling condition. Imaging and biochemical markers (biomarkers) are important diagnostic and research tools that can assist with this challenge.

## Introduction

Osteoarthritis (OA) is the most common disorder of synovial joints, such as the knees, hips, and hands, and the most significant source of societal cost in older adults ^1^. Conservative estimates from the Global Burden of Disease 2010 study suggest that of all the chronic health conditions, hip and knee OA was ranked as the 11th highest contributor to global disability and 38th highest contributor to disability-adjusted life years (DALYs) ^2^. It is estimated by the World Health Organization that by the year 2050, 130 million people will suffer from OA worldwide and 40 million will be severely disabled, highlighting the significant societal burden that this serious disease will present ^3^.

Joint damage in OA may occur through repeated and excessive mechanical loading on the joint and the cumulative impact of low-grade inflammation over time, or through injury, sustained during the life course ^4^. Loss of articular cartilage structure and function is one of the major hallmarks of OA ^5– 7^. The gradual articular cartilage degradation, bone changes, low-grade inflammation and synovitis cause: joint pain, anatomical changes, and swelling; impairing mobility and reducing quality of life ^8^. OA symptoms are debilitating and, as well as causing physical impairment, can affect the psychosocial wellbeing of patients, paving the way for novel psychological interventions ^9^. In the absence of disease-modifying treatments, the management of OA must be tailored to the individual and focus on core treatments, including self-management and education, exercise, and weight loss as relevant ^1^.

Recent evidence suggests that OA is a heterogeneous and multifaceted disease with multiple molecular and clinical phenotypes ^10^. The ability to classify patients into different groups and identify patients with relatively rapid OA progression can significantly transform OA clinical trials and enhance their efficiency. This approach has been applied to rheumatoid arthritis (RA) ^11^ and is a well-established segmentation and stratification strategy for identifying “clinicopathobiologic clusters” (another term that may be used to describe molecular endotypes) ^12^ and developing targeted therapeutics for asthma ^13^. Here we provide a narrative review of advances in understanding the molecular and clinical phenotypes of OA over the last 3 years. We also discuss biomarkers that have potential to be useful for molecular phenotyping of OA patients ^14^. We can learn a great deal from the molecular phenotyping approaches that are currently used in other disease areas and apply them in basic research and clinical development for OA.

## Clinical and molecular endotypes of osteoarthritis

Emerging evidence over the last few years suggests that OA is a heterogeneous and multifaceted disease with multiple molecular and clinical phenotypes ^10, 14^. Identifying phenotypes of OA is an important research priority because it allows us to gain a better understanding of the pathways and mechanisms that may be involved in each distinct phenotype and target them more effectively using a variety of preventive and treatment strategies ^15^.

In the field of asthma research, the concept of phenotypes has already paved the way for a comprehensive molecular and cellular classification of different forms of asthma ^16^. However, in this field, the clinical phenotype was defined as the presentation of the disease in an individual and focuses on the presentation of the disease rather than the molecular mechanisms underlying it. Whereas, the molecular endotype, alludes to the pathogenesis at the molecular and/or cellular level, ignoring its presentation. See Box 1 and Box 2 for further details. Insights from the asthma field may be deployed in the future, to benefit the OA research community, as efforts to create and disseminate similar, consensus-based definitions in the OA field are currently ongoing.

Box 1. **Definition of endotype**

In biology, endotype may be defined as a specific molecular pathway that explains the observable properties of a phenotype. In medicine, an endotype is a subtype of a disease or condition, which is defined by a distinct functional or pathobiological mechanism. Endotype implies the presence of a well-defined molecular mechanism and is distinct from a phenotype (see Box 2). Molecular endotypes may be defined by specific cells or biomarker molecules in blood or other body fluids.

Box 2. **Definition of phenotype**

In biology, phenotype can also be defined as “observable properties of a living organism that are produced by the interactions of the genotype and the environment”. In medicine, a phenotype may be any observable characteristic or trait of a disease, such as morphology, development, biochemical or physiological properties, or behavior, without any implication of a molecular mechanism or pathway. In clinical medicine, patients with common characteristics are often grouped together to guide therapy and improve management. Phenotyping is very useful for studying, diagnosing, and treating any disease, particularly those that are inflammatory and degenerative. However, at the present time, clinical phenotypes are the most common method of subgrouping. There are several problems with clinical phenotyping. First, there may be no specific tests or biomarkers that identify a particular phenotype compared to another phenotype. Therefore, diagnosis is usually left to the judgment of the clinician or researcher and may not be definitive. Second, there are not consensus definitions for specific subgroups. For this reason, poorly understood subgroups may go undiagnosed and untreated and lumped together with others.

## Clinical phenotypes of osteoarthritis

**Figure 1.**
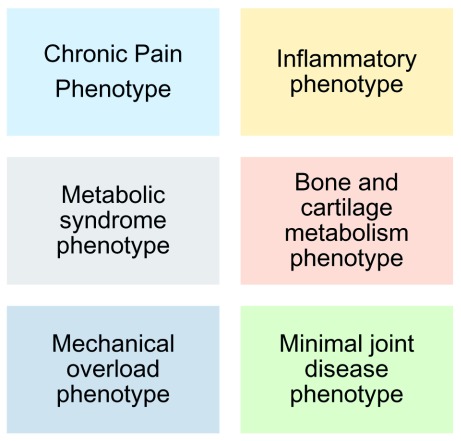
Clinical phenotypes of knee osteoarthritis. Clinical phenotypes of knee osteoarthritis, as originally identified by Dell’Isola *et al*. ^17^.

A systematic review of the literature conducted by Dell’Isola and colleagues in 2016 examined the current evidence for the existence of groups of variables which may point towards the existence of distinct clinical phenotypes in the knee OA population ^17^. They reviewed a total of 24 studies and, through qualitative synthesis of the available evidence, they found evidence for six distinct knee OA phenotypes (see Figure 1). They found that 84% of the subjects examined could be classified into six major phenotypes and that 12% of the total could be classified into an “inflammatory” category according to synovitis/effusion scores from the MOAKS (Magnetic Resonance Imaging [MRI] Osteoarthritis Knee Score).

A number of trials of anti-IL-1 agents did specifically focus on OA patients with associated synovitis. A recent phase II clinical trial of the anti-IL-1α/β dual variable domain immunoglobulin lutikizumab (ABT-981) in patients with knee OA and synovitis showed limited improvement in the WOMAC pain score and no improvement in synovitis. Trials of other IL-1 inhibitors in knee OA showed similar results ^18^. ABT-981 was also tested in erosive hand OA in a phase IIa, placebo-controlled, randomized trial. Again, neither pain nor imaging outcomes improved compared with placebo ^19^. These outcomes are in clear contrast with ample data showing that IL-1β is a critical pro-inflammatory cytokine mainly activating the MAPK (mitogen-activated protein kinase) pathway, eventually resulting in elevated levels of matrix metalloproteinases that can cause cartilage matrix protein degradation ^20^. This might indicate that IL-1β alone may be less pivotal in driving OA pathobiology than initially hypothesized. Yet other factors might also be relevant, such as the disease stage of the study subjects and suboptimal outcome measures. Moreover, selecting particular molecular endotypes rather than clinical phenotypes may still help identify patient subgroups that could benefit from targeted anti-inflammatory strategies such as IL-1β blockade.

In a subsequent paper published two years later, Dell’Isola *et al*. used data from the Osteoarthritis Initiative (OAI) to classify a sample of individuals with knee OA into pre-defined groups characterized by specific variables that may be linked to different disease mechanisms and compare these phenotypes for demographic and health outcomes ^21^. They selected 599 patients from the OAI database to conduct the study. Findings from existing studies and open source data were used to identify cut offs of key variables for each phenotype. After each of the three steps of the selection, classified patients were discounted from subsequent stages. Those who fell into more than one phenotype were assigned a separate ‘complex knee OA’ group. Using the OAI dataset, the authors were able to allocate phenotypes for 84% of cases with an overlap of 20%. Disease duration was shorter in the minimum joint disease while they found that the chronic pain phenotype included more women (81%). This subsequent study demonstrated the feasibility of using a classification system for knee OA subjects and placing them in distinct phenotypes based on subgroup-specific characteristics ^21^.

An expert working group assembled by the European Society for Clinical and Economic Aspects of Osteoporosis, Osteoarthritis, and Musculoskeletal Diseases (ESCEO) and the European Union Geriatric Medicine Society (EUGMS) suggested possible patient profiles in OA. Herreo-Beaumont *et al*. discussed the existence of four distinct phenotypes, including biomechanical, osteoporotic, metabolic, and inflammatory, and proposed that further characterization of these phenotypes would help to properly stratify patients with OA in clinical trials ^22^.

A review article published in 2014 by Karsdal *et al*. proposed five potential phenotypes, including mechanotransduction, hormonal, metabolic, auto-inflammation, and genetic subtypes. Their motivation for reviewing the literature was to propose the idea of finding the “right patients” for the “right drug” in OA. They suggested key drivers of the disease and their speculated impact on the rate of disease progression, which influences the duration of clinical trials. They also proposed that the drivers of OA progression may be divided into at least three different categories: bone, cartilage, and inflamed synovium, the key tissues that make up the synovial joint. These tissues may represent different disease subgroups; alternatively, they may represent the predominant and tissue-specific pathologies during a particular stage of disease. Optimal therapy may be considered the ability to detect and target each of these stages or subgroups of disease. Although the review by Karsdal *et al*. did not take a systematic approach, it did highlight methods for possible segregation of OA patients that would allow the identification of patient subtypes, particularly the small population of OA patients whose disease is driven by inflammation, proposing that this group offers pharmaceutical companies the “low hanging fruit” and may be ideally suited for personalized healthcare and for the development of smarter and more targeted therapies ^23^.

## Molecular endotypes of osteoarthritis

**Figure 2.**
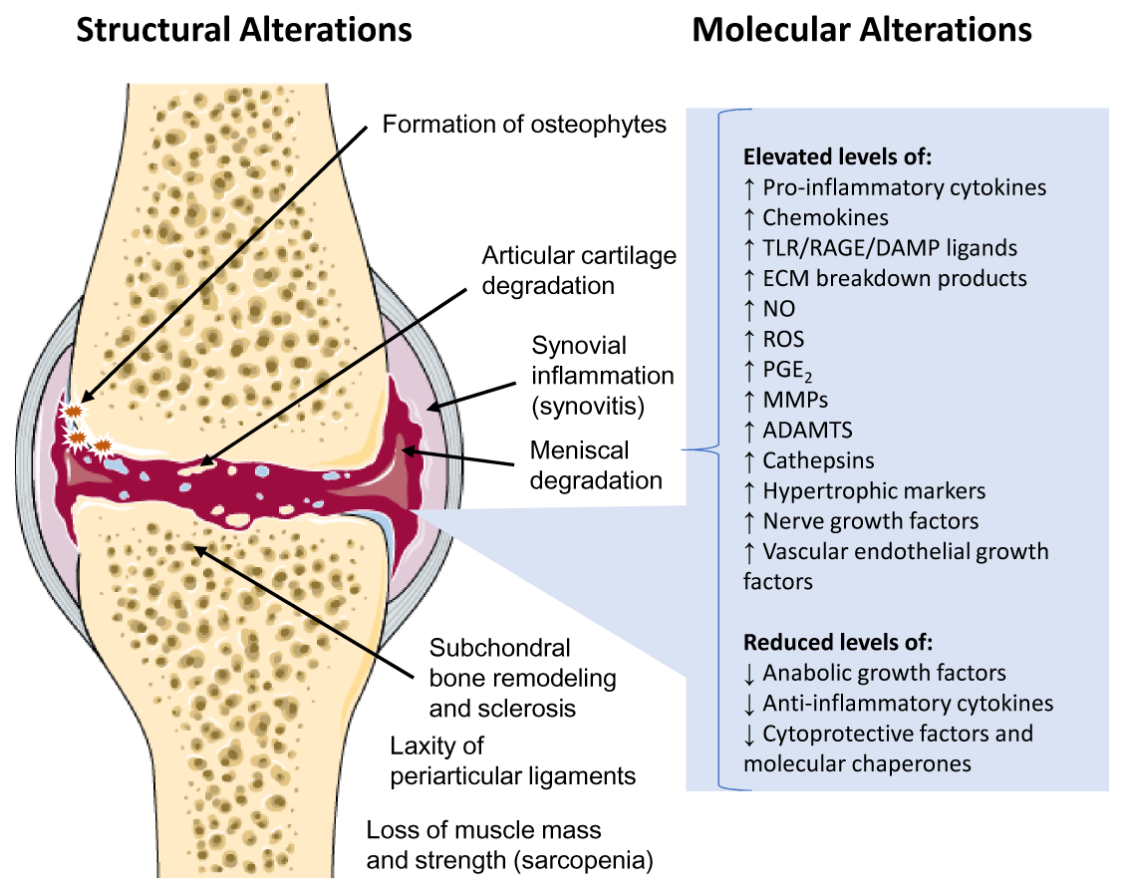
Summary of the major structural and molecular alterations in osteoarthritis. Molecular alterations in the micro-environment of chondrocytes and changes in the structure of the extracellular matrix (ECM) alter the behavior and physiology of chondrocytes. ADAMTS, a disintegrin and metalloproteinase with thrombospondin motifs; MMPs, matrix metalloproteinases; NO, nitric oxide; PGE _2_, prostaglandin E2; RAGE, receptor for advanced glycation end-product; ROS, reactive oxygen species; TLR, Toll-like receptor.

As explained above, molecular OA endotypes are disease subtypes that are defined by distinct molecular mechanisms and signaling pathways. When investigating mechanisms of disease development and progression, we should recognize that different clinical OA phenotypes may consist of overlapping molecular endotypes that may be identified by the presence of specific cells or biomarker molecules in blood or synovial fluid, which is the most suitable proximal tissue fluid for assessing the molecular and cellular taxonomy of OA subtypes. We already know a great deal about the structural and molecular alterations that occur in the cartilage ECM and within chondrocytes in OA cartilage ( Figure 2).

The structural alterations that are seen in the articular cartilage, synovium, subchondral bone, and other peri-articular tissues using imaging techniques such as radiography, MRI ^24^, ultrasonography ^25^, and computed tomography (CT) are preceded by molecular and cellular changes that occur many years before structural changes come to light ^26^. Indeed, the “molecular phase” of the disease can remain latent for decades ^27^. The most recent definition of OA published by the Osteoarthritis Research Society International (OARSI) states “the disease manifests first as a molecular derangement (abnormal joint tissue metabolism) followed by anatomic, and/or physiologic derangements (characterized by cartilage degradation, bone remodeling, osteophyte formation, joint inflammation and loss of normal joint function)” ^28^. The updated definition also mentions the role of pro-inflammatory pathways of innate immunity which implicate the innate immune system in OA pathogenesis ^29^. The molecular alterations in early OA can be studied using epigenomics, transcriptomics, proteomics, and metabolomic and lipidomic platforms ^30^ and by monitoring changes in the chondrocyte secretome ^31^. More extensive alterations in the cartilage ECM occur in the intermediate and later stages of the disease, and these can be monitored using assays of biochemical markers derived from ECM breakdown ^32– 34^.

**Figure 3.**
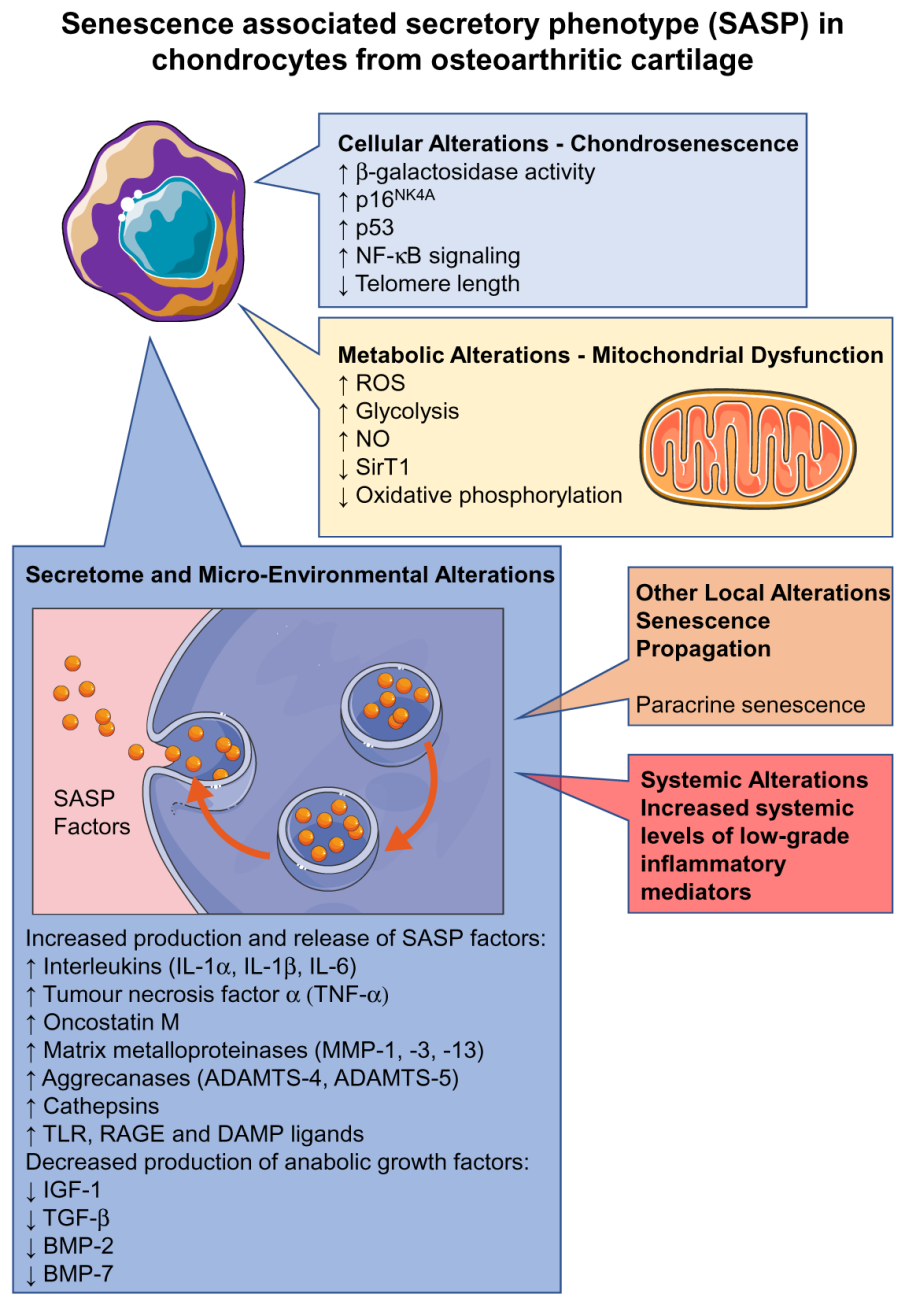
Senescence-associated secretory phenotype (SASP) in chondrocytes from osteoarthritis cartilage, highlighting phenotypic alterations at the cellular level in cells ^35, 36^. BMP, bone morphogenetic protein; DAMP, damage-associated molecular pattern; IGF-1, insulin-like growth factor-1; NF-κB, nuclear factor κB; NO, nitric oxide; RAGE, receptor for advanced glycation end-product; ROS, reactive oxygen species; TGF-β, transforming growth factor-β; TLR, Toll-like receptor.

There are a number of early molecular alterations that occur within chondrocytes and in their surrounding micro-environment. These are important changes and can provide clues about molecular endotypes. Recent research on the senescence-associated secretory phenotype (SASP) in chondrocytes from OA cartilage has revealed phenotypic alterations at the cellular level, including chondrosenescence ^35, 36^, metabolic alterations at the mitochondrial and glycolytic levels, and extensive molecular changes in the secretome and the micro-environment of OA cartilage (detailed discussion of these changes is beyond the scope of this review, but they are worth mentioning and summarizing in Figure 3).

## The inflammatory osteoarthritis phenotype

Discussion of all the putative endotypes of OA is beyond the scope of this review. However, the inflammatory phenotype is worth discussing in more detail, as it has implications for the development of therapeutic strategies that have failed and future therapies that may be more targeted. We have known about the existence of an inflammatory phenotype of OA for more than 40 years (since 1975) but have not paid careful attention to the historic literature. In a paper published over 40 years ago, George E. Ehrlich described a cohort of predominantly menopausal females who presented with a deforming and inflammatory OA, some of whom went on to develop inflammatory changes characteristic of RA but clearly did not have RA ^37, 38^. This classic study is as relevant now as it was when it was originally published. Inflammation is important, but it is not always present at all OA stages. If we take a cross-section of a large population of OA patients at all stages of disease progression, we will find only a small proportion that will display inflammatory features. This does not mean that inflammation is not important in OA; it simply highlights the temporal aspects of its manifestation. Clearly, inflammation is an important molecular endotype in OA. Synovial inflammation (synovitis) is believed to contribute to the pathophysiology and symptoms of OA through increased local production of pro-inflammatory cytokines, chemokines, and mediators of joint tissue damage ^39^. Therefore, treating the most aggressive forms of synovial inflammation holds great promise for symptom and structure modification in OA ^40^. However, in order to do this, we must possess sensitive tools to detect aggressive synovitis. Inflammation of the synovial membrane may occur in both the early and the late phases of OA and is associated with mononuclear cell infiltration of the synovial membrane and alterations in the adjacent cartilage that are similar to those seen in RA ^41, 42^. From a clinical perspective, Hoffa’s synovitis in the knee and the formation of osteophytes are relatively strongly associated with the presence of knee pain. We know that the presence of Hoffa’s synovitis and osteophytes in any region of the knee joint is significantly associated with the presence and severity of pain in OA ^43^. MRI and Doppler ultrasonography can be used to diagnose synovitis and identify the most aggressive forms in the knee ^44^. The sensitivities for detecting effusion and synovitis using ultrasonography are excellent ^44^. Correlating ultrasound imaging with histopathological findings and MRI findings gives us the ability to identify those patients with aggressive synovitis. Ultrasound also allows us to assess the late-stage OA changes in the knee ^45^. Therefore, we might already have the tools; we simply need to use them for patient stratification. We also have the opportunity to combine imaging biomarkers with biochemical markers to develop combination biomarkers that can define each molecular endotype, allowing us to start defining the underlying endotypes.

## Discussion

**Figure 4.**
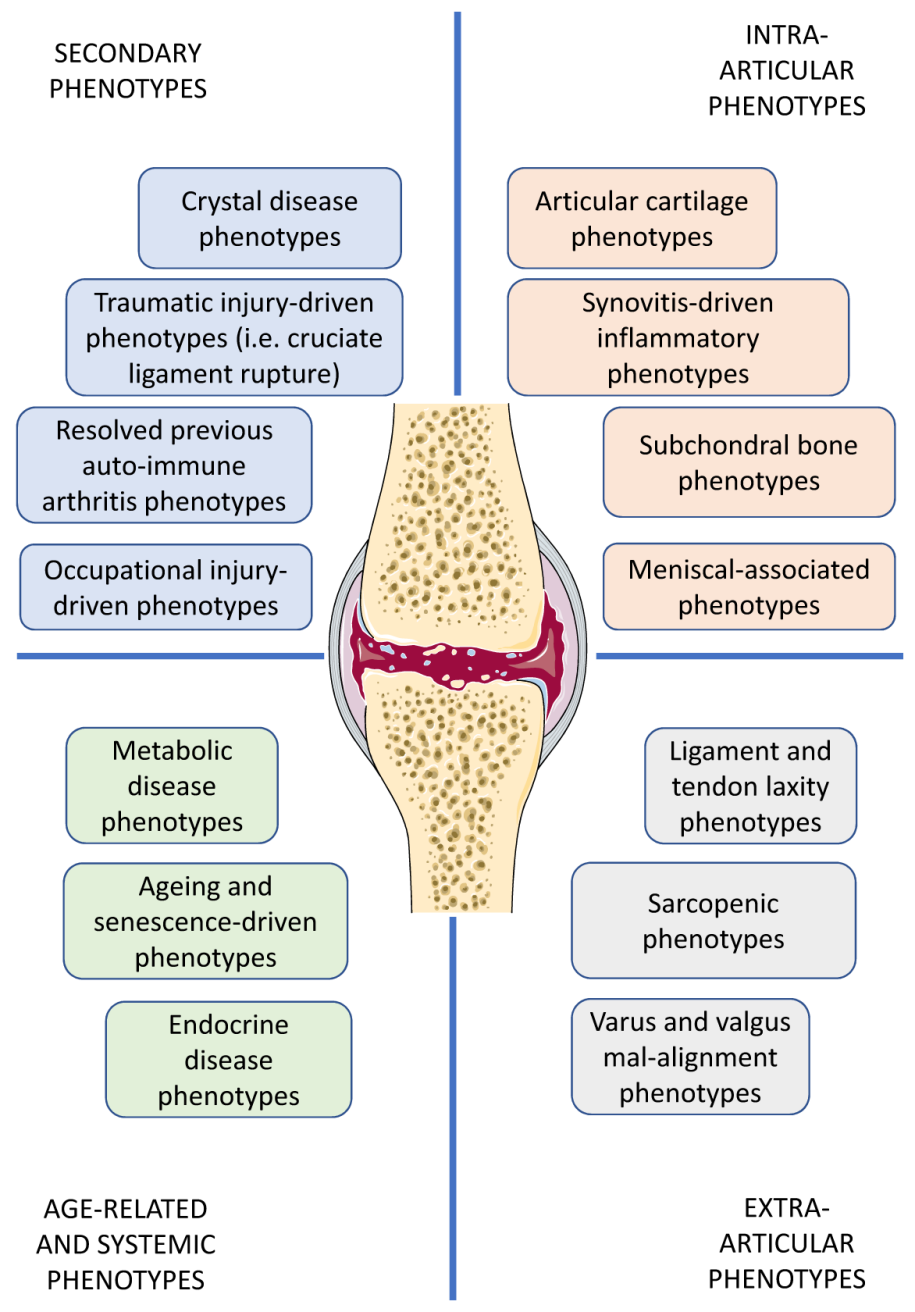
Diverse clinical osteoarthritis phenotypes.

**Figure 5.**
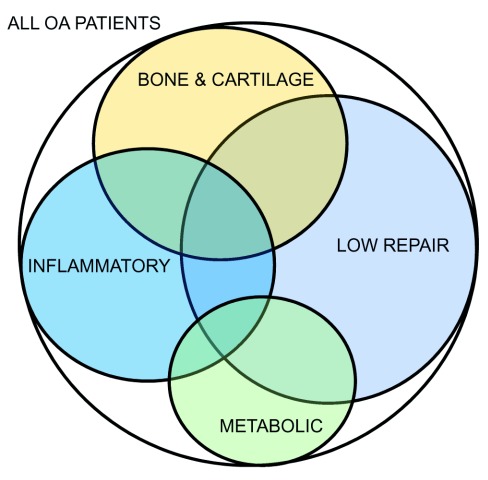
Venn diagram illustrating diverse overlapping molecular endotypes of osteoarthritis. Venn diagram illustrating diverse overlapping molecular endotypes of osteoarthritis (OA).

It is very likely that multiple molecular and clinical phenotypes of OA exist and that they are important to take into account in OA research and clinics. The first goal is to identify the clinical phenotypes and clearly define their constituent molecular endotypes. This is a challenging task, as clearly many of these are interconnected and mechanistically linked ( Figure 4 and Figure 5). For example, a low-repair phenotype may be an overarching age-related phenotype that may overlap with multiple different phenotypes, resulting in a set of people with more rapidly progressing OA and/or in need of more aggressive treatment targeting multiple mechanisms. Likewise, a mechanical phenotype may provoke several molecular mechanisms, such as a cartilage phenotype, that later becomes inflammatory, which may be particularly important within an obese phenotype (a high-fat or high-adiposity phenotype). Clearer definition of the molecular endotypes is needed, and much more research is needed to achieve this. A better understanding of the underlying molecular endotypes will allow us to define the clinical phenotypes more clearly and develop biomarker panels that can predict disease progression and determine which patients may have a better capacity for joint repair. We may even be able to distinguish between patients who will benefit from earlier total joint replacement surgery and those who can keep their joint for a longer time. This can lead to significant savings in healthcare management, enhanced clinical trials, and more effective OA drug development pipelines.

The inflammatory phenotype of OA is the most interesting one from our perspective. The published evidence suggests that synovial inflammation is associated with progressive joint failure, at least in a subgroup of patients, and therefore some OA patients may benefit structurally from an anti-inflammatory intervention ^46^. Indeed, the failure to identify and select (molecular) subgroups of OA patients who will benefit most from anti-inflammatory treatments may be the reason why clinical trials targeting inflammation have failed so far ^40^. Synovitis is qualitatively and quantitatively different in OA and RA, but there are significant inflammatory and fibrotic findings observed in the synovium of some OA patients ^41^. Synovitis in OA is characterized by macrophage infiltration and activation and is also associated with osteoblast activation and fibrosis ^47^. Synovitis in OA also predicts structural progression and varies in parallel with pain. Synovial inflammation is particularly pronounced in a subset of knee OA patients, and those with a prominent inflammatory profile are more likely to respond to NSAIDs ^48^. Therefore, pain profiling may also be used to assess patient responses to other drugs such as methotrexate, biologics, steroids, and other emerging anti-inflammatory treatments.

Comparison of RA and OA using microarray profiling of human joint fibroblast-like synoviocytes has revealed important similarities and differences between synovitis in these joint diseases ^49^. More detailed examination of fibroblast-like synoviocytes (FLSs) in OA and RA will reveal more about the mechanistic differences between synovitis in these two diseases, but we already know that NF-κB signaling and tumor necrosis factor α (TNF-α) are also important in synovitis in OA ^49^.

It is important to use standardized techniques and platforms to establish biomarkers of the inflammatory OA phenotype. The laboratory of Virginia Kraus is already making progress in this area by analyzing synovial fluid (SF). A recent study aimed to identify a SF biomarker profile characteristic of individuals with an inflammatory OA phenotype. The authors used a high-sensitivity multiplex immunoassay, Myriad Human InflammationMAP® 1.0, which included 47 different cytokines, chemokines, and growth factors related to inflammation to identify a subset of six SF biomarkers specifically related to synovial inflammation in OA. They also correlated the biomarker profiles to radiographic features and symptom severity. The six OA-related SF biomarkers were specifically found to be linked to indicators of activated macrophages and neutrophils. These results provide the first panel of biomarkers to characterize an inflammatory OA phenotype. This panel of biochemical markers may be measured easily and could serve as the basis for therapeutic targeting of a subset of individuals at high risk for knee OA progression ^50^.

The challenges of studying phenotypes offer the OA research community and pharmaceutical companies involved in developing disease-modifying OA drugs (DMOADs) new and exciting opportunities for innovation. The challenge is to develop sensitive diagnostics that can diagnose the disease in the molecular and pre-radiographic stages and determine which mechanisms are at play. The use of soluble biomarkers will allow more specific targeting of the underlying mechanisms by identifying the key tissues involved (i.e. cartilage, subchondral bone, and synovium). Therefore, panels of biochemical markers will need to be established to define the molecular endotypes of OA. This approach has the capacity to treat and target the disease more effectively. However, finding biomarkers with the sensitivity and specificity to achieve this is a challenging problem in OA biomarker research and currently cannot be done with the currently available biomarkers in our toolbox. A systematic review published by van Spil and colleagues nine years ago concluded that “none of the current biochemical markers are sufficiently discriminating to aid diagnosis and prognosis of OA in individuals or limited numbers of patients or performs so consistently that they could function as an outcome in clinical trials” ^51^. The conclusions of this study are still valid today.

## Conclusions

We have learnt a great deal about clinical and molecular endotypes from research into asthma ^52^. Now that asthma is known to be a heterogeneous inflammatory disease composed of molecular endotypes, it has been possible to develop targeted therapies as well as biomarkers capable of identifying treatment-responsive patients. We are already using biomarker assays and applications in the research and clinical trial settings to identify clinical and molecular endotypes of severe asthma ^53^. The OA research community is several years behind the field of asthma research, but we have the opportunity to identify and apply imaging and biochemical markers to better define the clinical and molecular endotypes of OA. Selecting and targeting of subjects in OA clinical trials will be greatly facilitated by gaining a better understanding of molecular endotypes. This will allow clinical trial designers to recruit subjects who have a high likelihood of responding, and potentially progressing, in a typical clinical trial time period (within 1–2 years in the case of OA) and can aid preclinical decision-making as well as pharmaceutical companies by demonstrating that a drug is successful ^27^. However, it is important to note that the subgroups and phenotypes may not segregate clearly and cleanly if we recruit a much older population of OA patients with multiple co-morbidities into clinical trials. It is envisaged that OA patients with a specific molecular endotype present themselves within multiple clinical phenotypic clusters. Some of these phenotypic clusters are likely to overlap, especially in older patients with multiple co-morbidities.
